# The Potential Therapeutic Role of Mesenchymal Stem Cells-Derived Exosomes in Osteoradionecrosis

**DOI:** 10.1155/2021/4758364

**Published:** 2021-12-02

**Authors:** Yuetian Li, Xinyue Wang, Yu Pang, Shuangcheng Wang, Meng Luo, Bo Huang

**Affiliations:** ^1^West China School of Stomatology, Sichuan University, Chengdu 610041, China; ^2^State Key Laboratory of Oral Diseases, and General Dentistry, West China Hospital of Stomatology, Sichuan University, Chengdu 610041, China

## Abstract

As one of the most serious complications of radiotherapy, osteoradionecrosis (ORN) seriously affects the quality of life of patients and even leads to death. Vascular injury and immune disorders are the main causes of bone lesions. The traditional conservative treatment of ORN has a low cure rate and high recurrent. Exosomes are a type of extracellular bilayer lipid vesicles secreted by almost all cell types. It contains cytokines, proteins, mRNA, miRNA, and other bioactive cargos, which contribute to several distinct processes. The favorable biological functions of mesenchymal stem cells-derived exosomes (MSC exosomes) include angiogenesis, immunomodulation, bone regeneration, and ferroptosis regulation. Exploring the characteristic of ORN and MSC exosomes can promote bone regeneration therapies. In this review, we summarized the current knowledge of ORN and MSC exosomes and highlighted the potential application of MSC exosomes in ORN treatment.

## 1. Introduction

Osteoradionecrosis (ORN) is regarded as the most destructive complication of radiotherapy [1, 2], which mainly manifests as chronic spontaneous pain, dysphagia, facial deformation, and other symptoms [3]. It seriously affects the quality of life of patients and even leads to death [4]. The first clinical evidence of ORN radiotherapy was reported in 1922 [5]. Using modern treatment techniques, such as intensity-modulated proton therapy, the estimated incidence of ORN has dropped to 2–5% [6–10]. However, ORN has the highest incidence in oral cancer radiotherapy, as high as 78% [11]. The incidence of the mandibular is significantly higher than that of the maxilla, mainly due to the higher blood supply of the upper jaw bone [12]. ORN is irreversible and can last for decades. However, there is still no gold standard treatment or consensus guidelines.

**Figure 1:**
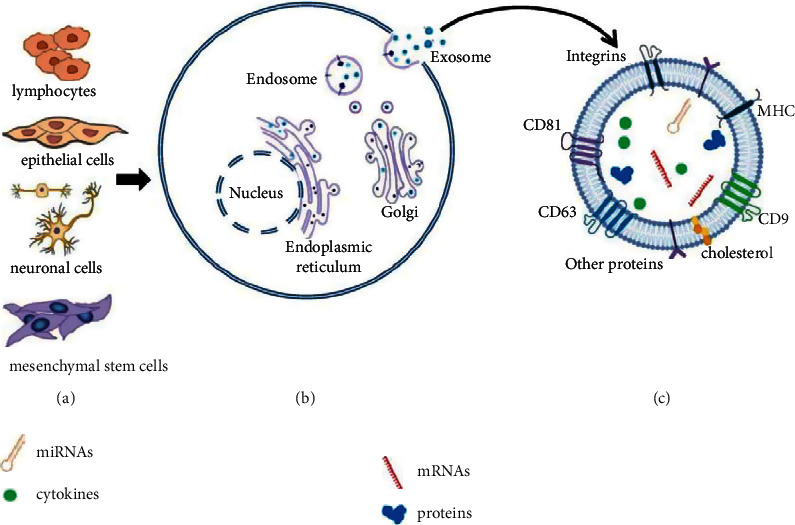
Characteristics of exosomes. (a) Exosomes derived from almost all types of cells. (b) Exosomes originating from an endocytic compartment and secreted from intracellular endosomes into extracellular space. (c) Exosomes are vesicles with a phospholipid bilayer membrane. The exosomes contain some biomarkers, such as CD9, CD63, CD81, and integrins, MHC, cholesterol, and other proteins on the surface. The exosomes also contain miRNAs, mRNAs, cytokines, and some proteins in the lumen. MHC, major histocompatibility complex.

Exosomes belong to a category of extracellular vesicles, with a diameter of 40–160 nm (an average of 100 nm) [13] and a density of 1.13–1.19 g/ml [14]. They are membrane-bounded phospholipid vesicles with a cup-shaped structure derived by all eukaryotic cells [15, 16] (Figure 1). These cells secrete exosomes through regulatory processes such as endocytosis, fusion, and efflux [17].

There are surface markers on the exosome membrane, such as CD63 [18–21], CD9, CD81 [22], and other transmembrane proteins. Exosomes contain many bioactive cargos, including cytokines, lipids, mRNAs, and more than 170 miRNAs and 304 proteins [13]. The contents of exosomes change dynamically and are related to the cell type and state. Recipient cells can take up the exosomes through diverse markers on the membrane to perform different functions [13]. Due to the rich sources, simplicity, safe, low immunogenicity, and other advantages, exosomes have become a hot spot in current research studies [23–27].

Among the potential providers of exosomes, such as epithelial cells [28], mast cells [29], dendritic cells [30], lymphocytes [31–33], and neuronal cells [34], mesenchymal stem cells (MSCs) have been widely concerned as seed cells in the field of tissue engineering and regenerative medicine. MSC exosomes are the primary product of MSCs [35]. Retaining similar biological characteristics and functions as MSCs, MSC exosomes are more stable and easier to preserve [36]. According to a report, MSC exosomes have enormous potency in repairing tissue lesions. They can promote the repair of damaged endometrium in intrauterine adhesion through the TGF-*β*1/Smad pathway [25]. They can change the immune environment to promote myocardial repair [37]. They can also promote skin regeneration and wound healing by accelerating angiogenesis, fibroblast proliferation, and collagen deposition [38–40]. In diabetic rats, exosomes derived from MSCs pretreated with atorvastatin can accelerate wound repair by promoting angiogenesis via the AKT/eNOS pathway [41].

**Figure 2:**
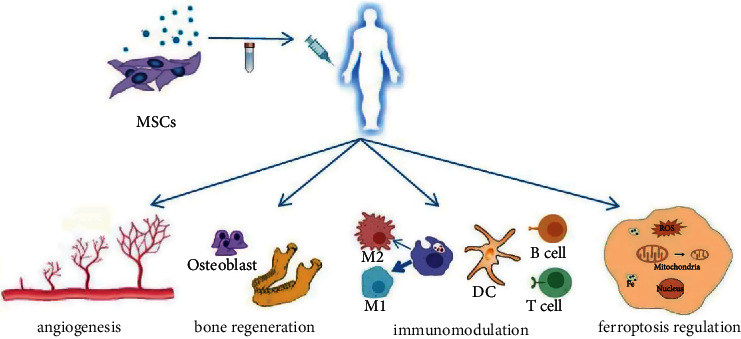
Therapeutic effects of MSC exosomes on ORN. Exosomes isolated from MSC and transferred to body. MSC exosomes exert their therapeutic effects on ORN through their angiogenesis, immune regulation, bone regeneration, and iron death regulation abilities.

MSC exosomes have the ability of angiogenesis, immunomodulation, bone regeneration, and ferroptosis regulation, which provides novel insight for the treatment of ORN (Figure 2). Therefore, this review will discuss the latest pathogenesis of ORN and the therapeutic mechanism of MSC exosomes. We also discuss the advantages and challenges of exosomes' clinical application.

## 2. Pathophysiology of ORN

ORN refers to the bones that cannot heal for more than three months and have no persistent tumors after being irradiated [42, 43]. Clinical signs and symptoms vary with the stage of ORN, including mucosa ulceration and necrosis [44], trismus [45], and suppuration [46]. Pain, anesthesia, halitosis, and dysgeusia are neurological symptoms when ORN occurs in the oral [42]. With the development of ORN, it becomes difficult to speak, masticate, and even open mouth [47–49]. Radiography, computed tomography (CT) scans, and orthopantomogram (OPT) are recommended to detect ORN according to the degree of bone lesions (one of its most typical pathological changes) [5]. However, the characteristics of the image are uncorrelated with the severity of ORN [50].

To clarify the pathogenesis of ORN, different scholars have proposed different hypotheses [51–56]. The first is the radiotherapy-trauma-infection theory [51], in which bacteria invade the jaw bone through the wound, cause chronic infection disease, and lead to ORN [48]. It was the basis of the popular antibiotic therapy for ORN. However, this theory had been questioned because no microorganisms had been found in deep lesions [52]. With the advancement of microbial detection technology, the presence of the deep microorganisms in radionecrotic mandibles was detected by DNA hybridization, suggesting that the theory may still be reasonable, and the role of anaerobic infection in ORN may be essential [57].

The second is the three-hypo theory based on vascular injury and immune dysfunction. After radiation, the hypoxic, hypocellular, and hypovascular state in the bone leads to vascular injury and immune dysfunction, causing chronic nonhealing wounds and ORN [52]. This hypothesis was once considered to be the most likely to explain the ORN mechanism [58]. For decades, hyperbaric oxygen (HBO) therapy based on it has been the standard and conservative choice for the treatment of ORN [59]. However, the development of HBO therapy based on this three-hypo hypothesis is still controversial [60–62]. Annane et al. demonstrated that patients with overt mandibular ORN did not benefit from HBO therapy in a randomized, double-blind, and placebo-controlled trial [61]. Forner et al. found that only minor stem cells were mobilized in head and neck irradiated patients treated with HBO [62]. Since there are few studies on postoperative radiotherapy for head and neck cancer, the effect of HBO on ORN and its specific mechanisms need to be further explored.

The third is the reactive oxygen species (ROS) theory, in which ROS caused endothelial cell damage in ORN [53, 63]. The occurrence of ORN was related to the ischemia caused by vascular embolism [54, 55] and the imbalance of bone regeneration caused by hypovascularity [64]. In addition, some studies have suggested that the radiation injury of osteoclasts occurs earlier than vascular changes, which may be the initial event in the development of ORN [48, 65–67].

The fourth is the mainstream radiation-induced fibroatrophic theory, in which the pathogenesis of ORN is divided into three stages. The first stage is the initial prefibrotic stage. Radiation-induced ROS and chemokines attract leukocytes to the injury sites, triggering an acute inflammatory response through ROS produced from macrophage and leading to endothelial cell damage [5, 63, 68]. The next stage is the constitutive organized stage. Due to the loss of the vascular endothelial barrier, cytokines such as FGF-*β*, TGF-*β*1, tumor necrosis factor-*α* (TNF-*α*), and interleukins (IL) result in the accumulation of fibroblasts and transdifferentiation of fibroblasts into myofibroblasts (MFB) [69–71]; The last stage is the late fibroatrophic stage. Hypoxic, hypocellular, and hypovascular environments can lead to bone fragility, and changes in the local metabolic environment can lead to ORN [53]. The key factor of this theory is the generation of MFB [72]. MFB proliferates rapidly and produces a large amount of extracellular matrix protein and collagen, which disrupts the balance of synthesis and degradation in the radiated tissue. As a result, fibrous tissue replaces the bone matrix, leading to the occurrence of ORN. In the meantime, the combination of pentoxifylline (PTX) and vitamin E for antioxidation and antifibrosis therapy has shown efficacy in clinical trials [73–76], which points out the direction for exploring new therapies.

Recently, ferroptosis has been discovered as an iron-dependent form of nonapoptotic cell death, providing a new possible theory for ORN [56, 77, 78]. Triggered by excessive lipid peroxidation, ferroptosis has morphological, biochemical, and genetic characteristics different from apoptosis [79, 80]. One of its representative characteristics is smaller mitochondria with condensed mitochondrial membrane densities [81]. During radiotherapy, ionizing radiation generates ROS and induces the expression of long-chain acyl-CoA synthetases 4 (a lipid metabolism enzyme), which leads to lipid peroxidation and ferroptosis [82]. In cancer cases, some small molecules promote ferroptosis and inhibit tumor cells by synergizing with radiation and inhibiting glutathione peroxidase 4 [83]. However, excessive ferroptosis also occur in normal cells, ischemia-reperfusion injury, kidney failure, neurodegeneration, and other diseases [56]. If ferroptosis occurs in the osteoblasts, osteoporosis and osteonecrosis will appear [84]. Exosomes derived from mouse vascular endothelial cells can reverse osteoporosis by inhibiting osteoblast ferroptosis [84]. However, there are insufficient clinical trials and basic experiments to prove the relationship between ferroptosis and ORN.

## 3. Traditional Treatments of ORNs

Based on the degree of bone and soft tissue damage, ORN consists of four stages (stage 0, stage I, stage II, and stage III) [60]. Different stages of ORN require distinct treatment protocols [85].

Surgical treatment has been widely used in ORN. Surgery includes removal of small sequestrum, ostectomy, radical resection, and flap reconstruction [85]. According to a review, the most common option for mandible reconstruction was a fibula-free flap with plenty of vessels to provide sufficient blood supply [86]. However, its relatively large wound and slow recovery are serious shortcomings.

In addition to surgery, there are six types of conservative therapies for ORN. They are debridement, HBO therapy, PTX and tocopherol (vitamin E), chlorhexidine, ultrasound therapy, growth factor, and MSC therapy. However, each of them has its shortcomings and can only be combined with other treatments.

Eliminating all bones that are no longer vascularized can prevent long-term infection and inflammation, thereby facilitating subsequent therapies [87]. According to recent evidence, HBO therapy was not recommended for ORN at any stage [61, 62, 88, 89]. PTX and vitamin E can prevent radiation-induced fibrosis (RIF) in patients with ORN through a synergistic effect [90–92]. A phase II trial showed that PTX, tocopherol, and clodronate (together referred to as PENTOCLO) helped 89% of patients to recover within 14 months [93]. Although PENTOCLO has a positive impact on the treatment of early ORN, stage II and III cases require surgery for assistance [94]. Chlorhexidine is a commonly used drug for the treatment of ORN since it can prevent infection and promote wound healing [95]. Chlorhexidine can be used as a bactericide against Gram-positive and Gram-negative microorganisms and some yeasts [96]. A study showed that with curettage and 0.12% chlorhexidine flushing as the main treatment, exposed bone closure occurred in 50% of cases, confirming the clinical effectiveness of chlorhexidine [97]. Ultrasound therapy can promote angiogenesis for revascularization of ORN [98–100]. However, due to the lack of further research, therapeutic ultrasound can only be used as an experimental option in clinical trials [85]. Due to its ability to regulate cytokines, growth factors and MSC therapy are other potential choices [85]. The plasma with growth factors-Endoret is beneficial to the vascularization and epithelialization of ORN [101]. The combination of rat MSCs and bone morphogenetic protein-2 (BMP2) is effective in the ORN treatment [102]. This feature of MSCs provides evidence for the potential therapeutic capability of the exosomes derived from MSCs.

Although conservative therapies can treat some early ORN, the cure rate is only 28.6%, combined surgery is required to obtain better effects, and the recurrence is possible [103]. Therefore, it is necessary to study a new treatment.

## 4. Therapeutic Effects of MSC Exosomes on ORN

### 4.1. Angiogenesis

Angiogenesis and vascularization play important roles in bone regeneration after radiation. Promoted by a variety of endogenous proangiogenic factors, including vascular endothelial growth factor (VEGF), hepatocyte growth factor (HGF), stromal-derived factor-1 (SDF-1), platelet-derived growth factor (PDGF), fibroblast growth factor (FGF), and epidermal growth factor (EGF) [104, 105], endothelial cells successively form buds, capillaries, and vessel networks [36]. Exosomes loaded with miRNA-7b, -9, -21, -26a, -27a, -210, -378, -195–497 cluster, -675–126 [106], -132 [107], -135b-5p, and -499a-3p show positive effects on angiogenesis [106–110]. Further studies have shown that the noncoding RNA cargos play essential roles in regulating angiogenesis by accommodating proangiogenic factors.

Recent studies have revealed that MSC exosomes with different contents can promote angiogenesis through various signaling pathways (Table 1). Exosomes secreted by MSC enhance angiogenesis through the Jagged 1 and Notch signaling pathway under the stimulation of hypoxia-inducible factor-1*α* (HIF-1*α*) [111]. Exosomes secreted by human bone marrow MSCs promote angiogenesis through the Akt/mTOR signaling pathway under the stimulation of dimethyloxalylglycine (DMOG) [112]. Exosomes secreted by human-induced pluripotent stem cells (hiPSC-MSC) enhance angiogenesis through the PI3K/Akt signaling pathway in endothelial cells [113, 114]. CD63^+^ exosomes secreted by bone marrow MSCs transported Wnt3 protein exteriorly to enhance angiogenesis [115]. Exosomes secreted by human placenta-derived MSCs (hP-MSCs) can enhance the angiogenic effects of HUVECs through increasing VEGF and miR-126 under the stimulation of nitric oxide (NO) [116].

The vascular injury through different mechanisms can also be prevented or even reversed by MSC exosomes. Exosomes play a key role in repairing DNA double-strand breaks and alleviating oxidative damage [117]. After exposure to MSC exosomes, the apoptosis caused by radiation-induced DNA damage in vascular endothelial cells is reduced [118].

### 4.2. Immunomodulation

According to the radiation-induced fibroatrophic theory, radiation-induced endothelial injury leads to necrosis and tissue ischemia in the prefibrotic stage and constitutive organized stage [53]. The released free radicals and chemokines attract white blood cells to the injury site and cause inflammation [68].

Since the immune disorder is one of the pathogeneses of ORN, MSC exosomes may become a potential treatment for ORN due to their immunomodulation capability in bone and cartilage tissue [36]. MSC exosomes exert kinds of anti-inflammatory function through immunomodulation [119–122]. First, MSC exosomes induce macrophages to shift from the M1-like to the M2-like phenotype [123]. The former is a classic proinflammatory cell type, and the latter is known for its anti-inflammatory responses [124, 125]. Exosomal miRNA-146 [123], miRNA-34 [126], and miRNA-181a [127, 128] can reduce the M1-related cytokines, such as IL-6, IL-12, and TNF-*α*, and enhance the M2-related cytokines, such as IL-10 and TGF-*β*, by promoting M2 polarization of macrophages [129, 130]. MSC exosomes also play the immunoregulatory role on osteogenesis by decreasing M1 phenotype markers of macrophage [131]. MSC exosomes loading with Wnt could activate Wnt/*β*-catenin signaling on target cells and alleviate radiation-induced bone loss [132]. Wnt/*β*-catenin signaling has been implicated in M2 macrophage polarization [133]. Second, MSC exosomes transport metallothionein-2, which causes inflammation reduction in a macrophage-dependent mechanism [129], participates in NO-mediated osteogenic pathways in osteoblasts [134]. Third, MSC exosomes mediate the acquisition of an immune tolerogenic phenotype in mature dendritic cells (DCs) [135]. Then, the tolerogenic DCs promote naïve CD4^+^ T cells to differentiate into Treg cells by secreting a variety of anti-inflammatory factors [135]. Fourth, MSC exosomes decrease lymphocyte proliferation [135] and serve as conveyors of the immunosuppressive effect on B lymphocytes [136]. In addition, the number of CD8^+^ T cells and the ratio of CD8^+^ T cells to CD4^+^ T cells in the peripheral blood were both restricted in certain conditions [137].

Some studies have proved the anti-inflammatory effects of MSC exosomes in bone and cartilage tissues [138–140]. Exosomes derived from adipose-derived MSCs can reduce the production of inflammatory mediators, such as TNF-*α*, IL-6, PGE2, and NO, to alleviate joint osteoarthritis (OA) [138]. Exosomes derived from human bone marrow MSCs can promote cartilage regeneration by inhibiting TNF-*α*-related collagenase activity [139]. They can also inhibit macrophage activation and chondrocytes apoptosis to treat joint damage [140].

To our knowledge, TGF-*β*1 and ROS are thought to play a more important role in radiation-induced fibrosis [141–144]. ROS can upregulate the expression of several fibrogenic genes by activating HIF-1*α* and releasing TGF-*β*1 [145]. It seems that we could come to reasonable speculation. MSC exosomes may slow down the fibrosis process in ORN development through immunoregulation. However, these conjectures require further study to confirm.

### 4.3. Bone Regeneration

Osteoblasts (OBs), derived from MSCs, account for 4–6% of osteocytes. The main function of OBs is to deposit calcium salts and form the bone. MSC exosomes can regulate the osteogenic differentiation of MSCs and the proliferation of OBs by using miRNAs to affect the expression of OBs-related mRNAs [146] (Table 2).

MSC exosomes containing miR-29a and miR-29c induce the osteogenic differentiation of MSCs by increasing the expression of OBs-related miRNAs, such as miR-206, miR-196a, and miR-27a [147]. At different time points of the osteogenic differentiation of MSCs, the expression of miR-199b, miR-218, miR-148a, and miR-135b increased, and the expression of miR-221, miR-155, miR-885-5p, miR-181a, and miR320 decreased in MSC exosomes [149]. The differential expression of let-7, miR-218, miR-196a, and miR-118a in MSC exosomes can also stimulate MSCs to differentiate into osteoblasts [148]. Studies have found that miR-885-5p regulates BMP2-induced osteogenic differentiation [149, 159]. MSC exosomes also promote the proliferation of OBs through miR-122-5p and the MAPK signaling pathway [160, 161]. Some contents of MSC exosomes, such as miR-92a-3p and miR-140-5p, can alleviate OA by promoting chondrogenesis, enhancing chondrocytes migration, and suppressing cartilage degradation [157, 158].

In addition, MSC exosomes can promote the proliferation of bone marrow stem cells and reduce radiation damage by reducing cell apoptosis and DNA damage [162]. Transplantation of human MSCs can enhance mouse bone marrow production and megakaryocyte production [163]. Injection of MSC exosomes can protect cd92/2 mice from delayed fracture healing [164]. The miR-148a-3p in MSC exosomes can prevent the osteonecrosis of the femoral head by inhibiting the expression of Smad ubiquitination regulatory factor 1 (SMURF1) [151].

### 4.4. Ferroptosis Regulation

Ferroptosis is an iron-dependent form of nonapoptotic cell death and is a newly discovered potential mechanism for tumors treatment [56, 77, 78]. If there is pathological ferroptosis in OBs, osteoporosis and osteonecrosis will occur [84]. The release of iron from exosomes mediates ferroptosis resistance [165]. Prominin-2 is a lipid dynamics regulation protein. It promotes the formation of multivesicular bodies (MVBs) and exosomes containing ferritin, thereby transporting iron out of cells and preventing ferroptosis [166, 167]. Given that exosomes are involved in the ferroptosis resistance in tumor cells [168], they may alleviate ORN by affecting the ferroptosis resistance in osteogenesis-related cells. MSC exosomes have high biocompatibility and efficiency [169, 170]. This characteristic may provide a novel idea for improving ORN by regulating ferroptosis.

### 4.5. Exosomes and Tumor Radiotherapy

Many studies have shown that exosomes are closely related to tumor radiotherapy. Exosomes derived from MSCs increase the inhibitory effect of radiotherapy on tumor metastasis [171]. In prostatic cancer, exosomes mediate radiation-induced nontargeting effects [172]. Radiation-activated p53 can be transmitted away through exosomes [173, 174]. Breast cancer exosomes promote DNA damage repair responses after radiation by regulating the phosphorylation of checkpoint kinase 1 (Chk1) [175]. Since exosomes can increase radioresistance through the miRNA inside [176, 177], we speculate that MSC exosomes may alleviate ORN by increasing the radioresistance of healthy bone cells.

## 5. Discussion

The treatment plan of ORN is comprehensive according to patients' age, compliance, and hospital conditions. The basic principles should be formulated based on classification and stage [85].

Due to the subsequent high infection rate, HBO treatment is not the best option [178]. At present, the traditional method for the latter stage of ORN is surgery combined with conservative treatments [85]. Adjuvant drugs such as chlorhexidine [179], antibiotics [85], and analgesics [180] can only be in combination with other surgical treatments, such as removal of small sequestrum, marginal mandibulectomy, segmental mandibulectomy, radical resection, and flap reconstruction [85]. However, a simple and atraumatic method is needed to treat ORN. MSC exosomes are promising candidates for ORN therapy, mainly due to their unique biological properties and various physiological effects.

MSC exosomes have higher biocompatibility than MSCs and can easily avoid immune rejection when transferred to impaired tissues [181]. MSC exosomes can avoid their internal specific cytokines or miRNA from being degraded by enzymes and achieve a stable therapeutic effect [182]. MSC exosomes can simultaneously activate multiple signaling pathways, avoid genetic modification of target cells, and provide repeatable and predictable results with stable phenotypes [181]. MSC exosomes have many advantages over traditional bone grafting because they can combine with a variety of biomaterials to repair bone defects [183]. Based on the above advantages, MSC exosomes show beneficial prospects in the treatment of ORN.

However, the clinical application of MSC exosomes faces many challenges. First, due to the conditions for the production of exosomes, the contents of exosomes are relatively unstable [184]. For example, the miRNA profile of exosomes is significantly affected by ionizing radiation [185]. Second, there is no uniform standard for the identification, quantification, and purification of exosomes, which lead to diverse results in dose-dependent experiments and uncertain effects in clinical applications [36]. The International Society for Extracellular Vesicles (ISEV) recommended several methods for the separation of exosomes, such as differential centrifugation, size exclusion chromatography (SEC), immunoaffinity capture, and combinations of the above techniques [186]. However, the specific application scopes of each method still need to be illustrated and unified. Third, there is still a lack of methods to obtain high purity exosomes while ensuring sufficient yield. Studies suggested that culturing MSCs in scalable microcarrier-based three-dimensional cultures with tangential flow filtration can improve the productivity of MSC exosomes [187]. But more research studies are needed to translate this experiment discovery into clinical application. Finally, some roles of MSC exosomes remain unknown or inconsistent. Their various functions depend not only on the lipids, nucleic acids, and proteins inside but also on the molecules and particles on the surface [188]. Since only a small part of the roles has been explored, it is urgent to improve and innovate the research methods and to conduct in-depth research on contents and application methods of MSC exosomes.

## 6. Conclusions

Taken together, MSC exosomes play important roles in ORN through their ability to regulate angiogenesis, immunomodulation, bone regeneration, and ferroptosis. Although the clinical application of MSC exosomes faces many challenges, this promising field will still attract further explorations and provide a more theoretical basis and clinical treatment for ORN.

## Figures and Tables

**Table 1:** MSC exosomes promote angiogenesis through various signaling pathways.

Exosomes	Pathway/key molecule	Function	Experiment type	Reference
MSC exosomes derived from overexpressing HIF-1*α*	Jagged 1/Notch	Enhanced angiogenesis and capillary-like tube formation	In vitro	[111]
Exosomes derived from DMOG-stimulated human bone marrow MSCs	Akt/mTOR	Promoted angiogenesis in the critical-sized calvarial defect rat model	In vivo	[112]
iPS-MSC-Exos	PI3K/Akt	Enhanced the proliferation, migration, and tube-forming capacities of endothelial cells	In vitro	[113]
Exosomes from hiPSC-MSC	—	Enhanced angiogenesis and osteogenesis under osteoporotic conditions	In vivo	[114]
CD63^+^ exosomes derived from bone marrow MSCs	Wnt3 protein	Enhanced endothelial angiogenesis	In vitro	[115]
Exosomes released from hP-MSCs by NO stimulation	VEGF and miR-126	Enhanced the angiogenic effects of HUVECs	In vitro	[116]

**Table 2:** The expression of partial miRNA derived from exosomes and the effects on osteogenesis.

mi-RNA	Expression level	Function
miR-29a	Carried in MSC exosomes	Induce the osteogenic differentiation of MSCs [147]
miR-29c		
miR-206	The expression of osteoblast-related miRNA was significantly increased [147]	Promote the proliferation and differentiation of OB [147]
miR-27a		
miR-196a		Stimulate the differentiation of BMSCs into osteoblasts [148]
miR-218	Significantly upregulated in exosomes isolated from BMSCs culture [149]	Stimulate the differentiation of BMSCs into osteoblasts [148]
miR-199b-5p		Promote chondrogenic differentiation [150]
miR-148a-3p		Prevent the osteonecrosis of the femoral head by inhibiting SMURF1 [151]
miR-135b		Enhance chondrocyte proliferation by downregulating SP1 [152]
miR-221	Significantly lower expressed in individual exosomal samples over time [149]	Inhibit osteogenic differentiation of BMSCs via the IGF-1/ERK pathway [153]
miR-155		Suppress osteoblastic differentiation by targeting SIRT1 [154]
miR-181a-3p		Inhibit osteogenic differentiation of MCSs by targeting BMP10 [155]
miR-320c		Reduce the osteogenic potential of BMSCs through Runx2 [156]
miR-885-5p		Exert a negative regulatory effect on the osteogenic differentiation of BMSCs by inhibiting Runx2 [149]
miR-92a-3p	Reduced in the OA chondrocyte-secreted exosome [157]	Promote chondrogenesis and suppress cartilage degradation [157]
miR-140-5p	Derived from miR-140-5p-overexpressing synovial mesenchymal stem cells [158]	Enhance proliferation and migration of chondrocytes through the Wnt signaling pathway [158]

## Data Availability

No data were used to support this study.
